# A BERT-Based Generation Model to Transform Medical Texts to SQL Queries for Electronic Medical Records: Model Development and Validation

**DOI:** 10.2196/32698

**Published:** 2021-12-08

**Authors:** Youcheng Pan, Chenghao Wang, Baotian Hu, Yang Xiang, Xiaolong Wang, Qingcai Chen, Junjie Chen, Jingcheng Du

**Affiliations:** 1 Intelligent Computing Research Center Harbin Institute of Technology Shenzhen China; 2 Peng Cheng Laboratory Shenzhen China; 3 University of Texas Health Science Center at Houston Houston, TX United States

**Keywords:** electronic medical record, text-to-SQL generation, BERT, grammar-based decoding, tree-structured intermediate representation

## Abstract

**Background:**

Electronic medical records (EMRs) are usually stored in relational databases that require SQL queries to retrieve information of interest. Effectively completing such queries can be a challenging task for medical experts due to the barriers in expertise. Existing text-to-SQL generation studies have not been fully embraced in the medical domain.

**Objective:**

The objective of this study was to propose a neural generation model that can jointly consider the characteristics of medical text and the SQL structure to automatically transform medical texts to SQL queries for EMRs.

**Methods:**

We proposed a medical text–to-SQL model (MedTS), which employed a pretrained Bidirectional Encoder Representations From Transformers model as the encoder and leveraged a grammar-based long short-term memory network as the decoder to predict the intermediate representation that can easily be transformed into the final SQL query. We adopted the syntax tree as the intermediate representation rather than directly regarding the SQL query as an ordinary word sequence, which is more in line with the tree-structure nature of SQL and can also effectively reduce the search space during generation. Experiments were conducted on the MIMICSQL dataset, and 5 competitor methods were compared.

**Results:**

Experimental results demonstrated that MedTS achieved the accuracy of 0.784 and 0.899 on the test set in terms of logic form and execution, respectively, which significantly outperformed the existing state-of-the-art methods. Further analyses proved that the performance on each component of the generated SQL was relatively balanced and offered substantial improvements.

**Conclusions:**

The proposed MedTS was effective and robust for improving the performance of medical text–to-SQL generation, indicating strong potential to be applied in the real medical scenario.

## Introduction

Electronic medical records (EMRs) contain abundant medical information on patients and are usually stored in structured relational databases with multiple relational tables [1]. Using EMRs, patient data can be traced back over an extended period of time and by multiple health care providers. EMRs can help identify those who are due for preventive checkups, screenings, or vaccinations. They also can record whether a patient’s vital signs (eg, blood pressure, weight) fall within normal limits [2,3]. However, retrieving EMRs from databases may not be easy for medical experts. They usually lack specific training on using SQL to perform queries on relational databases. Even for experienced informaticians, it could be troublesome to deal with massive SQL queries from databases of different structures and applicable scenarios, especially if complex SQL grammars were involved. Therefore, automating the transformation of textual questions written in natural language into SQL queries has great potential to facilitate clinical information retrieval and improve the efficiency of medical diagnosis and treatment decisions.

**Figure 1:**
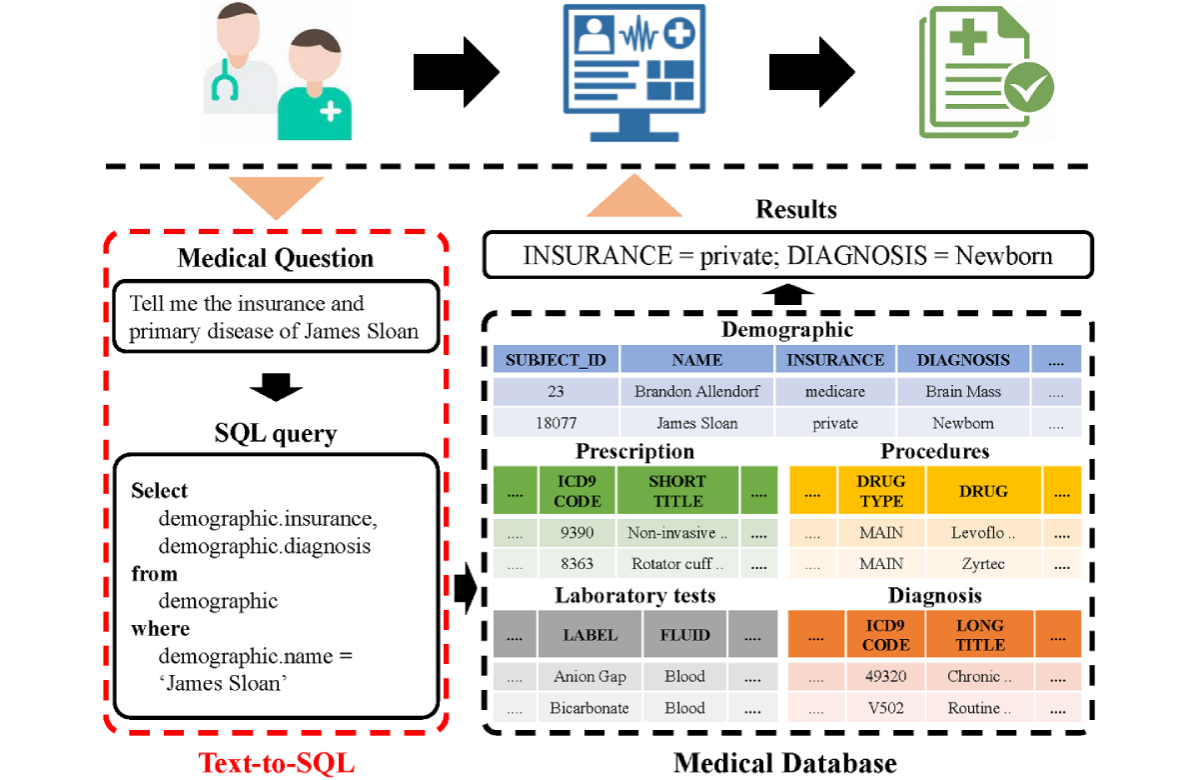
Application scenario of medical text–to-SQL generation.

Text-to-SQL generation [4,5] is the task of transforming natural language questions into SQL queries. As shown in Figure 1, given the medical textual question “Tell me the insurance and primary disease of James Sloan,” a text-to-SQL model can transform the question into a SQL query. It is then used to retrieve the corresponding EMR information that is stored in structured medical databases. This task has attracted widespread attention from different domains. The representative studies include automatic terminal information service [6-8] for a flight booking system, GeoQuery [9,10] for a US geography query, WikiSQL [5] for querying Wikipedia, and Spider [11] for realistic applications of several different domains. In many studies, the text-to-SQL generation is regarded as a task similar to natural language generation. Deep neural networks are often adopted as encoders and decoders (eg, the sequence-to-sequence [Seq2Seq] [12] framework with an attention [13,14] or copy mechanism) [15]. The input of the model is the textual question and the output is the SQL query that is viewed as an ordinary word sequence [16,17]. However, the same SQL query can be represented by multiple word sequences, which may affect the training effectiveness of Seq2Seq models. For example, the order of the 2 column names in the Select clause shown in Figure 1 may not influence the execution result of the query, but the Seq2Seq models may treat them as 2 different sequences. To solve this problem, several methods were proposed by incorporating the syntactical structure of SQL [18,19]. For instance, SQLNet [18] proposed a sketch-based sequence-to-set method. A generic sketch highly in line with the SQL grammar was first used and then it only needed to predict the slots in the sketch instead of generating the entire sequence in order.

Compared with other domains, corresponding explorations in the medical domain are insufficient. Due to the privacy requirements of medical data, a large-scale training corpus is still lacking. Furthermore, jargon and specialized phrases often occur in the medical text. They cannot be represented well by the models trained on other domains. But these terms are sometimes the key points of a medical question. In the limited relevant research, rule-based or those verified on small-scale datasets are most often found, such as methods of translating the medical questions into SPARQL Protocol and RDF Query Language (SPARQL) queries [20] and converting the clinical questions into EMR-dependent structured queries [21]. To push this forward, Yu et al [22] introduced a new criteria-to-SQL generation dataset for clinical trials. However, the targeted free text is quite different from other query text in terms of length and content. Wang et al [23] constructed the first large-scale text-to-SQL generation dataset, MIMICSQL, in the medical domain based on the widely used Medical Information Mart for Intensive Care (MIMIC III) dataset [24]. They also proposed a Seq2Seq-based model, Translate-Edit Model for Question-to-SQL (TREQS), to directly generate the SQL query for a given medical question by using the dynamic and temporal attention mechanism and controlled copying technique. But these works are preliminary explorations and do not integrate much intrinsic information related to the SQL itself (eg, the tree structure of SQL). Therefore, there is still much room left for further progress.

In this study, we propose a novel model for medical text–to-SQL generation named MedTS for the medical text–to-SQL generation task. First, the medical entities (ie, the table and column names) are recognized via schema linking. A pretrained Bidirectional Encoder Representations From Transformers (BERT) [25] model is then used as an encoder to enhance the question representation. The BERT-based encoder can exploit the relationship of entities between medical text question and database schema. Second, a grammar-based long short-term memory (LSTM) [26] decoder is adopted to generate the tree-structured intermediate representation instead of directly transforming a medical question into SQL query. It is in accordance with the chronological order of the syntax tree of SQL and can reduce the search space at each decoding step. Finally, according to the predefined set of context-free grammar, the intermediate representation is transformed into the corresponding SQL query. Experiments were conducted on the MIMICSQL dataset. We compared the proposed model with 5 competitor methods and further analyzed the performance of each component of the generated SQL query. An online system is accessible to better demonstrate the application of MedTS [27].

## Methods

### Dataset

We evaluated our proposed method on MIMICSQL [23], which is the first large-scale medical dataset for text-to-SQL generation task in the health care domain. The medical information in MIMICSQL is derived from MIMIC III. All of the medical information was first anonymized to protect patient privacy and then stored in 5 tables in the medical database (Figure 1), including demographic (Demo), laboratory tests (Lab), diagnosis (Diag), procedures (Pro), and prescriptions (Pres). The questions and corresponding SQL queries in MIMICSQL were automatically generated based on fixed templates [28]. Next, 8 freelancers with medical domain knowledge were recruited from a crowd-sourcing platform to validate the question as realistic and reasonable or rephrase the generated question. The information of the MIMICSQL dataset is summarized in Table 1. We adopted the same data partition as in the TREQS [23], in which all the question-SQL pairs were randomly split into training, validation, and test sets in the ratio of 0.8:0.1:0.1, respectively.

**Table 1:** The summary of the MIMICSQL dataset.

Type	Count
Patients, n	46,520
Tables, n	5
Columns in tables^a^, n	23/5/5/7/9
Question-SQL pairs, n	10,000
Template question length (in words), mean	18.39
Rephrased question length (in words), mean	16.45
SQL query length, mean	21.14
Aggregation columns, mean	1.1
Conditions, mean	1.76

^a^The 23/5/5/7/9 correspond to the numbers of columns in the Demographics/Diagnosis/Procedure/Prescriptions/Laboratory tests tables.

### Overview of the Proposed Method

Given a textual question *X*={*x*_1_,*x*_2_,*x*_3_,...} and the database schema, the goal of this work was to transform the textual question into a SQL query, while ensuring the SQL query retained the same semantic meaning as the textual question.

**Figure 2:**
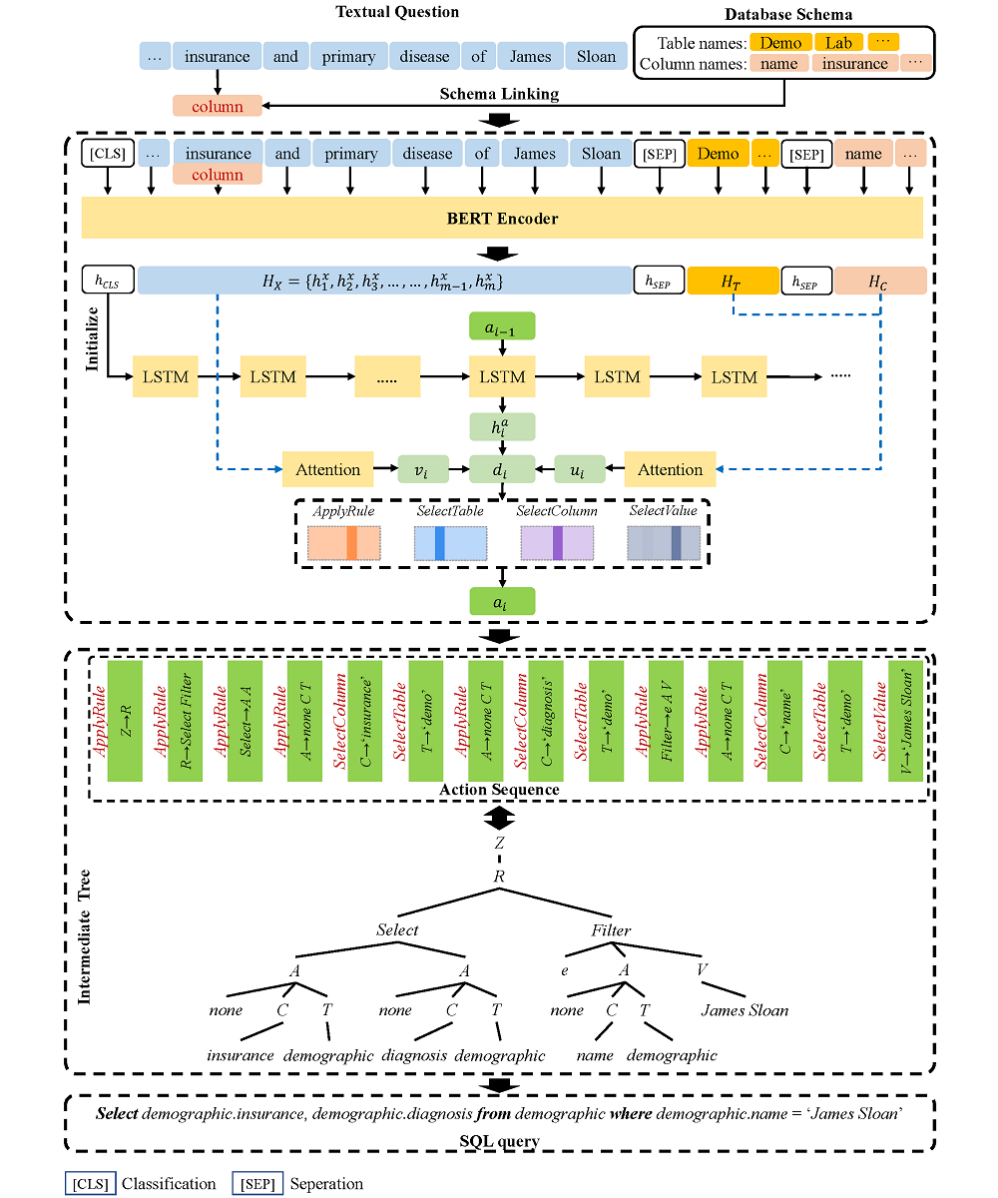
Overview of our proposed method of medical text–to-SQL task. LSTM: long short-term memory.

An overview of MedTS is shown in Figure 2. In the first step, schema linking recognized the database schema information and added corresponding linking marks into the question. Second, the textual question along with linking marks and the database schema information were fed into the pretrained encoder and grammar-based decoder to generate an intermediate representation. Third, the final SQL query was generated based on the intermediate representation.

### Schema Linking

Similar to the method in IRNet [19], the purpose of schema linking was to recognize the mentioned entities in the medical question and assign a linking mark, which referred to recognizing the column names and table names in the medical database. We enumerated all the *n*-grams (*n*∈[1,5]) in a question and arranged them in descending order based on the length. If an *n*-gram exactly matched a column name or was a subset of a column name, we marked this *n*-gram as a column. The recognition of a table followed the same way. If an *n*-gram was recognized as both a column and a table, we marked it as a column because the column mark has higher priority than the table mark. Once an *n*-gram was identified, we removed other n-grams that overlapped with it. By doing this, we obtained all the entities mentioned in the question. Once an entity was recognized and linked with a mark, it became a span and was encoded into one vector in the encoding process, such as the insurance recognized as a column in Figure 2.

### Attention-Based Encoder Using Pretrained BERT

After schema linking, we identified the entities and assigned linking marks. The given medical question *X* was transformed to [(*x*_1_,τ_1_),...,(*x*_m_,τ_m_)] where *x_i_* was the *i^th^* span and *τ_i_* was the mark of *x_i_* assigned during schema linking. If *x_i_* was not an entity, *τ_i_* was *None*. Let *C*={*c*_1_,*c*_2_,...} and *T*={*t*_1_,*t*_2_,...} denote the set of all column names and table names. In order to enhance the relationship between the question and database schema, we concatenated the question *X* and database schema [*C*,*T*] with special tokens, where one classification token [CLS] was used as the first token and several separation tokens [SEP] were used as separators of different information, as follows:



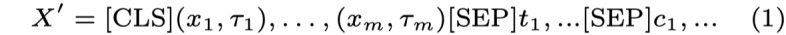



In this work, we first used a pretrained BERT as the encoder. The purpose was to convert the textual question with marks assigned by schema linking and database schema into hidden representations via the multihead attention mechanism [29]. Then, for each span, we took the average of the hidden representations of word and mark as the span representation. Last, through a fully connected pooling layer, we obtained the final hidden representations of question *H_X_*, column names *H_C_*, and table names *H_T_*. The nonlinear transformation of [CLS] representation *tanh*(*WH_CLS_*+*b*) was used to initialize the decoder, where *W* and *b* were trainable parameters.

### Tree-Structured Intermediate Representation

**Figure 3:**
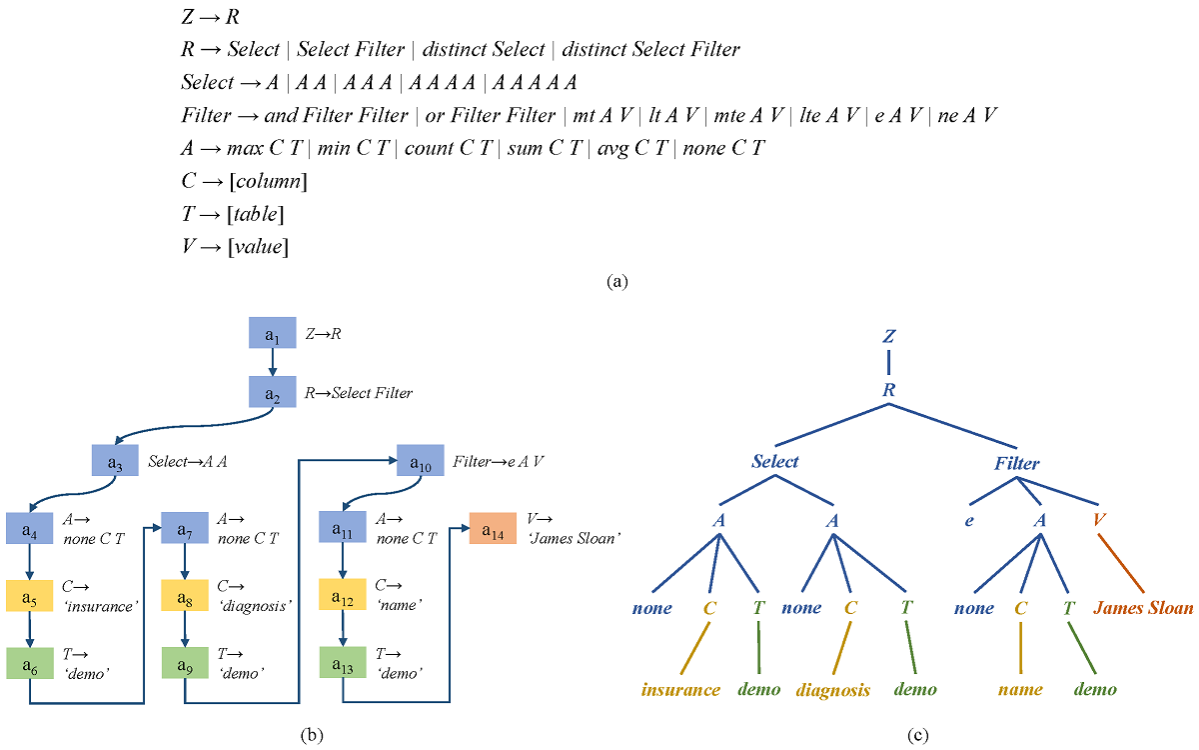
Example of tree-structured intermediate representation: (a) grammar rules that transform the SQL query into an abstract syntax tree, (b) example of the action sequence generated by the grammar-based decoder with 4 types of actions, and (c) intermediate tree constructed from the action sequence in b following the grammar rules in a.

Tree-structured intermediate representation refers to a syntax tree that bridges the question and the SQL query. It contains SQL information implicated in the textual question and could be transformed to a SQL query more intuitively due to the nature of the tree structure of SQL. Figure 3 demonstrates an example of generating an intermediate tree from an input question. Different from the previous works that used the grammar to assist the SQL generation [19,22], we designed the grammatical rules based on the MIMICSQL dataset. Specifically, we kept only the necessary rules occurring in the dataset to make the prediction more accurately and added a new rule for predicting the condition value.

To construct the intermediate tree from a SQL query, we first defined a set of grammar rules, as shown in Figure 3a. The intermediate tree starts from a root node *Z*. Since there are no complicated SQL components such as *Union* in this task, a single node *R* was directly attached to *Z*. Then, we attached a node *Select* or *Filter* under *R*, which was determined by the *Select* clause or *Where* clause, respectively. For the subtree of node *Select*, according to the number of columns in the *Select* clause, the same number of nodes *A* were attached to node *Select*. Each node *A* comprised an aggregation function node, a node *C*, and a node *T*. The aggregation function could be either of *none*, *max*, *min*, *count*, etc, while node *C* denoted the column name and node *T* denoted the table name. For the subtree of node *Filter*, it was determined by the conditions in the *Where* clause. If there was more than one condition, the corresponding number of *Filter* nodes would be attached. Next, for each *Filter* node, it attached a relational operator, a node *A*, and a node *V*. Relational operators include *more than* (*mt*), *less than* (*lt*), *equal* (*e*), etc. Node *V* denotes the condition value. The intermediate tree in Figure 3c was generated by the action sequence in Figure 3b following the grammar rules defined in Figure 3a. The generation process was in the depth-first, left-to-right order.

### Grammar-Based Decoder

The generation process of the intermediate tree was formalized into sequential applications of actions. The actions either applied a production rule on the derivation tree or produced a terminal node. According to the grammar rules, we defined 4 types of actions (ie, *ApplyRule*, *SelectColumn*, *SelectTable*, and *SelectValue*) and adopted the grammar-based decoding strategy [30,31]. *ApplyRule*(*r*) applied a production rule *r* to construct the skeleton of the intermediate tree, and the other 3 types of actions were designed to produce the terminal tokens. Thus, the goal of the decoder was to generate an action sequence *A* based on the outputs of the encoder. Formally, the decoding process was formalized as follows:



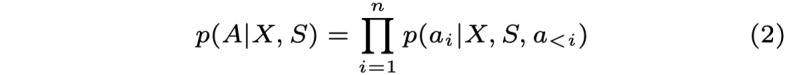



where *a_i_* was the action taken at time step *i*, *a*_<_*_i_* was the sequence of actions before *i*, and *n* was the number of total time steps of the whole action sequence.

The probability of selecting a rule *r* as the current action *a_i_* was calculated as follows:



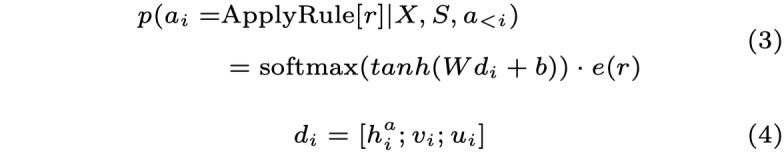



where 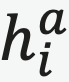 denoted the current hidden state of LSTM, *v_i_* and *u_i_* denoted the context vectors that were obtained by performing attention over *H_X_* and [*H_C_*;*H_T_*], *e*(*r*) was the one-hot vector for rule *r*.

The *SelectColumn* action was implemented via a memory-enhanced pointer network to select a column *c*, in which the memory was used to record the selected columns [32]. Once a column was selected, it was removed from the schema and recorded in the memory. The probability of selecting a column *c* was calculated as follows:



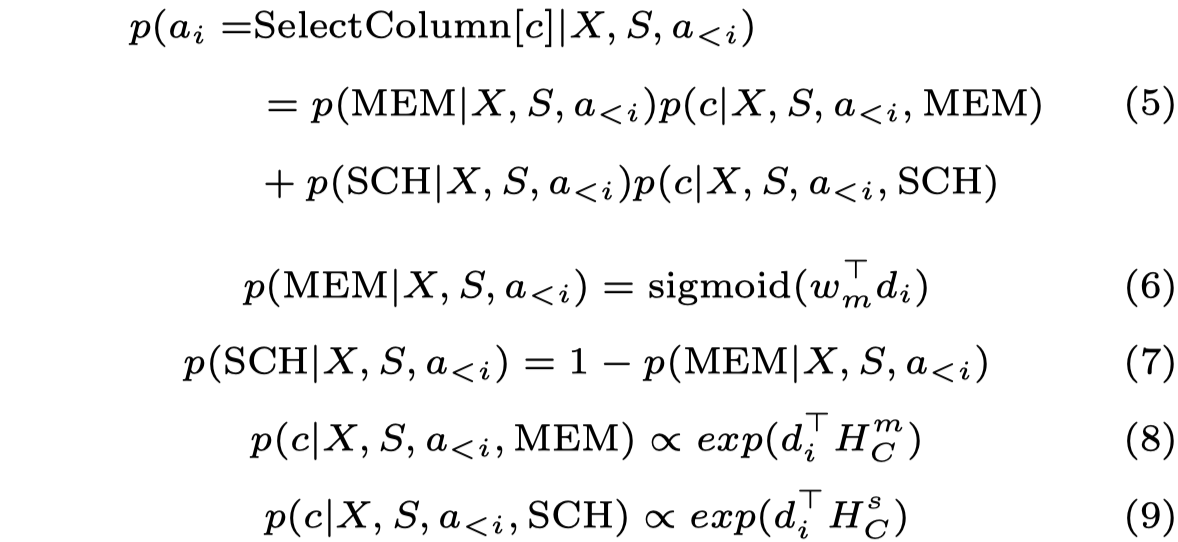



 where SCH denoted selecting from the schema, MEM denoted selecting from memory, and 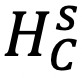 and 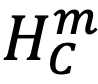 denoted the corresponding hidden representations of columns.

For the *SelectTable* action, we leveraged the relationship between columns and tables to prune irrelevant tables. Thus, the decoder predicted the table *t* that the selected columns belong to. The probability of choosing a table *t* was calculated as follows:



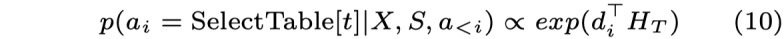



As for *SelectValue*, since the value was always mentioned in the textual question, the decoder extracted a condition value *v* by finding a start position and an end position from the question via 2 different pointer networks, respectively, as follows:



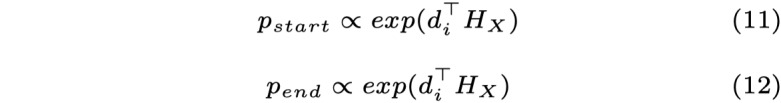



where *p_start_* and *p_end_* denoted the probabilities of the start and end positions.

Afterward, in order to keep the extracted value consistent with the value in the database, we also adopted the condition-value-recover technique proposed in TREQS [23] to find the most similar value in the look-up table content by computing the ROUGE-L (Recall-Oriented Understudy for Gisting Evaluation based on the Longest Common Subsequence) [33] score between them.

### SQL Query Generation

According to the grammar rules in Figure 3, when inferring a SQL query from an intermediate tree, we traversed the whole intermediate tree and mapped each node to the corresponding SQL component. The production rule applied on node *Z* denoted that it was just a single SQL query. The node *R* represented the start point. Following the child nodes of node *R*, we generated the skeleton of a SQL query, such as whether the SQL query had a *Select* clause or *Where* clause corresponding to the node *Select* and *Filter*, respectively. The node *Select* indicated how many columns the *Select* clause had. The node *Filter* indicated how many different conditions were in the *Where* clause. Based on the subtree of node *Select* or *Filter*, we filled in the details (ie, the aggregation function, relational operator, column name, table name, and condition value). The *From* clause was generated from the nodes of selected tables by identifying the shortest path that connected these tables in the schema.

### Experimental Settings

We adopted the pretrained uncased base BERT as our encoder, and the hidden size was set as 768. For the decoder module, the hidden size of LSTM was set as 300. The maximum length of the action sequence was set as 128. The size of the attention vector was set as 300. The coarse-to-fine framework [34-36] was used to model the generation process. The Adam optimizer [37] was adopted to train the model parameters for 100 epochs. The learning rate was set as 1e-06, and gradient clipping was used with a maximum gradient norm of 5.0. During training, we set the batch size as 8. The numbers of *ApplyRule*, *SelectColumn*, and *SelectTable* candidate actions were 24, 39, and 5 respectively. The size of the *SelectValue* candidate action was based on the length of the input textual question. We selected the model which achieved the best performance on the validation set. The MedTS was implemented with PyTorch [38] and trained on a Tesla V100 GPU (NVIDIA Corp). Our code has been shared on GitHub to facilitate other researchers [39].

We compared our proposed MedTS with 5 competitor methods. Seq2Seq [14] is an LSTM-based model with the attention mechanism, in which the SQL query is regarded as an ordinary word sequence. PtrGen [15] is a Seq2Seq-based pointer-generator network, which can directly copy the word from the input question to alleviate the repetition and out-of-vocabulary (OOV) phenomenon. SQLNet [18] is a sketch-based text-to-SQL model to avoid the order problems that occurred in the Seq2Seq model. Coarse2Fine [36] is a 2-stage neural architecture for text-to-SQL. A classifier is first used to obtain a rough sketch of the SQL query and then the details of SQL are filled in based on the input and the sketch individually. TREQS [23] is also a 2-stage text-to-SQL model, including an attentive-copying mechanism and condition value recovery mechanism.

All text-to-SQL methods were evaluated with 2 popular metrics [5], execution accuracy (*Acc_EX_*) and logic form accuracy (*Acc_LF_*), which are complementary to evaluate the quality of the generation of SQL queries.

- *Acc_EX_*=*N_EX_*⁄*N*, where *N* denotes the total number of question-SQL pairs and *N_EX_* denotes the number of SQL queries that can be executed and achieve the correct answers
- *Acc_LF_*=*N_LF_*⁄*N*, where *N_LF_* denotes the number of queries that match exactly with the ground truth of the SQL query

## Results

### Quantitative Evaluation

Table 2 provides the quantitative results on the validation and test sets. Seq2Seq achieved 0.103 *Acc_LF_* and 0.173 *Acc_EX_* on the test set. SQLNet performed better than Seq2Seq, since it considered the dependencies between the components of SQL query based on a graph derived from the sketch. But it was not easy to cover all the queries. PtrGen performed much better than SQLNet with 0.180 *Acc_LF_* and 0.292 *Acc_EX_* on the test set because it directly extracted words from textual questions to reduce the OOV words, especially when most values occurred in the original question. Coarse2Fine achieved decent performance since it incorporated the schema information into question encoding, but it was limited by the number of sketches and had difficulty handling more complex SQL. TREQS further improved the performance via several effective mechanisms, such as controlled generation and placeholder replacement. But it is just based on the Seq2Seq framework and did not consider the intrinsic structure information of SQL itself. Compared to all the methods mentioned above, MedTS achieved the best performance with 0.681 *Acc_LF_* and 0.880 *Acc_EX_* on the validation set and 0.784 *Acc_LF_* and 0.899 *Acc_EX_* on the test set, which outperformed the best competitor method by at least 29% and 27% in terms of *Acc_LF_* and *Acc_EX_*, respectively, on the test set.

**Table 2:** The logic form accuracy (*Acc_LF_*) and execution accuracy (*Acc_EX_*) of SQL query generated by various methods.

Methods	Validation		Test	
	*Acc_LF_* ^a^	*Acc_EX_* ^b^	*Acc_LF_*	*Acc_EX_*
Seq2Seq	0.092	0.195	0.103	0.173
SQLNet	0.086	0.225	0.142	0.260
PtrGen	0.181	0.325	0.180	0.292
Coarse2Fine	0.217	0.309	0.378	0.496
TREQS	0.562	0.675	0.556	0.654
MedTS	0.681	0.880	0.784	0.899

^a^*Acc_LF_*: logic form accuracy.

^b^*Acc_EX_*: execution accuracy.

### Performance on Each Component of SQL

In order to further analyze the generation result, we broke down the SQL queries into 5 components according to the SQL grammar structure, including aggregation operation, aggregation column, table, condition column along with its operation, and condition value. The experimental results are shown in Table 3. Since Coarse2Fine cannot handle multitable questions and is limited by table-aware assumption, its performance cannot be compared to other methods. Aggregation operation refers to the operations in the *Select* clause used to aggregate all the values of a column and return a single value, such as *Count*, *Sum*, *Avg*, etc. All methods except for Coarse2Fine achieved a very high accuracy of more than 97%. Aggregation column was the target column in the *Select* clause for the aggregation operation. MedTS outperformed other methods significantly by at least 5% and 12% on validation and test sets, respectively. Table was the target table in the *From* clause. Except for the Coarse2Fine, the other methods achieved similar accuracy. MedTS achieved the best performance. Condition column along with its operation represented the column and operation in the *Where* clause. Compared to the other competitor methods, MedTS achieved a large improvement by at least 8% on the test set. Condition value refers to the condition value in the *Where* clause. It was observable that the performance on condition value primarily played a vital role in the overall SQL generation performance. MedTS achieved improvement by at least 11% on the test set. In summary, the experimental results of MedTS on each component of SQL were relatively balanced and better, especially the performance on aggregation column and condition value.

**Table 3:** Accuracy of each component of SQL query.

Methods	Validation						Test				
	*Agg* ^a^ *_op_* ^b^	*Agg_col_* ^c^	*Table*	*Con* ^d^ *_c+o_* ^e^	*Con_val_* ^f^	*Agg_op_*		*Agg_col_*	*Table*	*Con_c+o_*	*Con_val_*
Coarse2Fine	0.321	0.313	0.321	0.260	0.214	0.524		0.490	0.528	0.448	0.413
Seq2Seq	0.978	0.872	0.926	0.471	0.174	0.970		0.696	0.892	0.565	0.296
SQLNet	0.994	0.939	0.933	0.722	0.080	0.989		0.873	0.941	0.749	0.140
PtrGen	0.987	0.917	0.944	0.795	0.236	0.987		0.830	0.926	0.824	0.235
TREQS	0.990	0.912	0.942	0.834	0.694	0.993		0.827	0.941	0.844	0.763
MedTS	0.994	0.988	0.971	0.893	0.785	0.991		0.985	0.951	0.919	0.851

^a^*Agg*: aggregation.

^b^*Op*: operation.

^c^*Col*: column.

^d^*Con*: condition.

^e^*c*+*o*: column and operation.

^f^*Val*: value.

### Ablation Study

We also conducted an ablation study to analyze the impact of schema linking as well as the use of different types of pretrained representations on question encoding and show the results in Table 4. When the schema linking was not used, the performance of MedTS dropped by 1.4% on *Acc_LF_* and 1.3% on *Acc_EX_* on the test set, which demonstrated the effectiveness of schema linking. The tested pretrained representations included a recurrent neural network (RNN)-based encoder (ie, BioWord2Vec [40]) and two BERT-based encoders (ie, ClinicalBERT [41] and BioBERT [42]). As shown in Table 4, the RNN-based encoder with pretrained BioWord2Vec performed far worse than the BERT-based encoder by at least 21.0% on *Acc_LF_* and 17.9% on *Acc_EX_* on the test set. We argue that the main reason is that the LSTM encoder cannot model the interaction of the entire sequence itself. As for the BERT-based encoders, we observed that the performance of ClinicalBERT was inferior to the others since it specializes in clinical notes that are obviously different from the natural language text. Compared to MedTS (with uncased base BERT), BioBERT achieved slightly better performance since it uses the medical literature for pretraining which is more beneficial to the representations of medical questions.

**Table 4:** The experimental results of the ablation study.

Methods			Validation				Test		
			*Acc_LF_* ^a^		*Acc_EX_* ^b^	*Acc_LF_*			*Acc_EX_*
**MedTS**			0.681		0.880	0.784			0.899
	w/o SL	0.669		0.870		0.773		0.887	
	w/ BioWord2Vec	0.472		0.690		0.501		0.644	
	w/ ClinicalBERT	0.556		0.771		0.634		0.784	
	w/ BioBERT	0.684		0.882		0.790		0.904	

^a^*Acc_LF_*: logic form accuracy.

^b^*Acc_EX_*: execution accuracy.

## Discussion

### Principal Findings

Our proposed model MedTS achieved the best *Acc_LF_* and *Acc_EX_* on the validation and test sets, with pretrained encoder and grammar-based decoder. The abstract syntax tree was introduced as the intermediate representation to bridge the gap between medical text and the SQL query. The primary outcomes of this study were (1) a new state-of-the-art model for medical text-to-SQL generation task was proposed and validated and (2) an online demonstration system with the capabilities of transforming the medical text to SQL query and further returning the query results was provided. Experimental results demonstrated that MedTS has great potential to help medical experts facilitate clinical information retrieval and improve the efficiency of decision-making for medical diagnosis and treatment.

### Model Performance

MedTS has the ability to capture the semantic relationship between words within textual questions and the dependency relationship between the text and database schema, benefitting from the multihead attention mechanism adopted by the pretrained encoder. It is difficult for competitor methods to obtain information as rich using the RNN-based encoder. Meanwhile, MedTS can effectively reduce the search space via the grammar-based decoding strategy, which predefines grammatical rules and introduces the tree-structured intermediate representation. Although several mechanisms were designed in the competitor methods to make the generated SQL query more accurate, they still view the SQL query as an ordinary word sequence and ignore the intrinsic structure characteristic of SQL itself, which makes them perform worse than MedTS.

### Case Study

In addition to quantitative evaluations, we conducted an extensive set of qualitative case studies on the test data to analyze the generated SQL query. We manually analyzed all 1000 text-SQL pairs in the test set. Among them, 784 generated SQL queries that were entirely consistent with the ground truth, and 115 generated SQL queries that were not identical to the ground truth in the logical form but also achieved accurate execution results. Most of them were caused by the different positions of the 2 tables connected by the *join* operation (eg, example 1 in Table 5). This phenomenon also explains why the quantitative evaluation results of *Acc_EX_* are higher than *Acc_LF_* in Table 2. In addition, 5 generated SQL queries were correct but considered wrong by the *Acc_EX_* because of the various orders of column in the *select* clause (eg, example 2 in Table 5). The remaining 96 pairs generated incorrect SQL queries. We grouped their errors into different categories from 2 perspectives: clause and element. The clauses included *select*, *join*, and *where*, and the elements included operator, table, column, value, and others. The statistical results are shown in Table 6. Note that there was no operator in the *join* clause. Similarly, since the value only presented in the *where* clause, the value error of *select* and *join* clauses was none. When there was a table error in the *where* clause, it was usually due to the wrong decision in the *select* or *join* clauses, so we did not count these types of errors again. The rest of the errors, such as more or less conditions, are grouped into other categories.

From the element’s perspective, we observed that the prediction errors of *column* and *value* account for the majority. From the perspective of the clause, more than 50% of clause errors were in *where* clauses, while most *where* clause errors were due to incorrect values or columns. Example 3 in Table 5 is a representative case of *where* clause error due to the incorrect value. The value of *expire_flag* is a numeric type in SQL but a text description in the question. Example 4 in Table 5 shows a case of *where* clause error due to the wrong column, in which the *admityear* and *dob_year* are semantically close, leading to the wrong choice. It was challenging to achieve high accuracy in these cases, since MedTS is based on the pointer network that selects terms from textual questions to generate SQL queries. The *operation* error means that the condition column and value in the *where* clause are correct but the operator is wrong, which may return completely opposite results, as shown by example 5 in Table 5.

**Table 5:** Five representative examples of qualitative case study.

Examples	
**Example 1**	
	Q^a^: Let me know the short title and ICD-9^b^ codes of diagnoses for patient John Gartman.
	G^c^: Select diagnoses.“icd9_code,” diagnoses.“short_title” from demographic inner join diagnoses on demographic.hadm_id = diagnoses.hadm_id where demographic.“name” = “john gartman”
	P^d^: Select diagnoses.“icd9_code,” diagnoses.“short_title” from diagnoses inner join demographic on diagnoses.hadm_id = demographic.hadm_id where demographic.“name” = “john gartman”
**Example 2**	
	Q: Tell me which primary disease the patient Walter Locher is suffering from and whether he is still alive or not.
	G: Select demographic.“expire_flag,” demographic.“diagnosis” from demographic where demographic.“name” = “walter locher”
	P: Select demographic.“diagnosis,” demographic.“expire_flag” from demographic where demographic.“name” = “walter locher”
**Example 3**	
	Q: Calculate the number of dead patients who were admitted to hospital before 2123.
	G: Select count (distinct demographic.“subject_id”) from demographic where demographic.“expire_flag” = “1” and demographic.“admityear” < “2123”
	P: Select count (distinct demographic.“subject_id”) from demographic where demographic.“expire_flag” = “0” and demographic.“admityear” < “2123”
**Example 4**	
	Q: How many American Indian/Alaska Native ethnic background patients were born before 2148?
	G: Select count (distinct demographic.“subject_id”) from demographic where demographic.“ethnicity” = “american indian/alaska native” and demographic.“admityear” < “2148”
	P: Select count (distinct demographic.“subject_id”) from demographic where demographic.“ethnicity” = “american indian/alaska native” and demographic.“dob_year” < “2184”
**Example 5**	
	Q: Find the minimum number of days of hospital stay for patients born before the year 2200.
	G: Select min (demographic.“days_stay”) from demographic where demographic.“dob_year” > “2200”
	P: Select min (demographic.“days_stay”) from demographic where demographic.“dob_year” < “2200”

^a^Q: textual question.

^b^ICD-9: International Classification of Diseases Clinical Modification, 9th Revision.

^c^G: golden truth.

^d^P: predicted result.

**Table 6:** Statistical analysis of error categories.

	Select	Join	Where	#Element Error (%)
Operator, n	9	—^a^	3	12 (10.6)
Table, n	8	6	—	14 (12.4)
Column, n	17	—	10	27 (23.9)
Value, n	—	—	44	44 (38.9)
Other, n	4	9	3	16 (14.2)
#Clause Error (%)	38 (33.6)	15 (13.3)	60 (53.1)	113 (100)

^a^Not applicable.

### Comparison With Prior Work

In the medical field, a few studies have focused on the text-to-SQL task, but most of them either proposed rule-based methods [20,21] or validated on the small-scale datasets [22]. Wang et al [23] constructed the first large-scale medical text–to-SQL dataset and proposed a neural model TREQS to undertake this task. However, TREQS focused on solving the OOV problem and condition value generation. Compared with the rule-based methods, our proposed model has better applicability and can be extended to other datasets. Compared with the previous neural models, our model adapts more advanced deep learning methods to this task and achieves the optimal experimental performance on a large-scale dataset.

### Limitations and Future Work

As discussed above, several problems are still to be solved, such as improving the accuracy of the *condition* *column* and *value* in the *where* clause, especially the gap between natural language description and the value stored in the database. In future work, we will continue to improve the accuracy and robustness of the model (eg, introducing more schema information such as the data type of column to achieve the goal of practical deployment). In addition, the form of question and SQL in MIMICSQL is relatively simple, which is not enough to cover various situations in the practical applications. Therefore, we plan to keep exploring different data forms for more practical scenarios, such as generating SQL queries containing more complex clauses.

### Conclusion

In this work, we proposed a medical text–to-SQL method named MedTS, which incorporates a BERT-based attention encoder to obtain schema-enhanced text representation and a grammar-based LSTM decoder to generate the intermediate action sequence before generating a SQL query. By introducing the intermediate representation, MedTS can reduce the search space during decoding and mitigate the mismatch problem between the medical question and the SQL query. Experiments on the MIMICSQL dataset demonstrate that MedTS substantially outperforms the state-of-the-art methods. Further analyses on each component of SQL query and the case study confirm MedTS’s effectiveness and robustness, demonstrating its strong potential.

## Abbreviations

*Acc_EX_*: execution accuracy
*Acc_LF_*: logic form accuracy
BERT: Bidirectional Encoder Representations from Transformers
EMR: electronic medical record
LSTM: long short-term memory
MedTS: medical text–to-SQL
MIMIC III: Medical Information Mart for Intensive Care III
OOV: out-of-vocabulary
RNN: recurrent neural network
Seq2Seq: sequence-to-sequence
SPARQL: SPARQL Protocol and RDF Query Language
TREQS: Translate-Edit Model for Question-to-SQL
ROUGE-L: Recall-Oriented Understudy for Gisting Evaluation based on the Longest Common Subsequence
